# Influence of treatment-related lymphopenia on the efficacy of immune checkpoint inhibitors in lung cancer: a meta-analysis

**DOI:** 10.3389/fonc.2023.1287555

**Published:** 2023-12-01

**Authors:** Ye Zhang, Cheng Huang, Shanqing Li

**Affiliations:** Department of Thoracic Surgery, Peking Union Medical College Hospital, Peking Union Medical College and Chinese Academy of Medical Sciences, Beijing, China

**Keywords:** lung cancer, lymphopenia, immunotherapy, survival, meta-analysis

## Abstract

**Background:**

Treatment-related lymphopenia (TRL) is common in patients with lung cancer, particularly in those with radiotherapy. However, the influence of TRL on the efficacy of immune checkpoint inhibitors (ICIs) for patients with lung cancer remains poorly understood. We performed a systematic review and meta-analysis to investigate the influence of TRL on survival of lung cancer patients on ICIs.

**Methods:**

In order to accomplish the aim of the meta-analysis, a comprehensive search was conducted on databases including PubMed, Embase, Cochrane Library, and the Web of Science to identify observational studies with longitudinal follow-up. The Cochrane Q test was employed to evaluate heterogeneity among the included studies, while the I^2^ statistic was estimated. Random-effects models were utilized to merge the results, considering the potential impact of heterogeneity.

**Results:**

Ten cohort studies with 1130 lung cancer patients who were treated with ICIs were included. Among them, 427 (37.8%) had TRL. Pooled results showed that compared to patients without TRL, patients with TRL were associated with poor progression-free survival (hazard ratio [HR]: 2.05, 95% confidence interval [CI]: 1.62 to 2.60, p < 0.001; I^2 = ^22%) and overall survival (HR: 2.69, 95% CI: 2.10 to 3.43, p < 0.001; I^2 = ^0%). Sensitivity analysis limited to patients with non-small cell lung cancer showed similar results (HR: 2.66 and 2.62, both p < 0.05). Moreover, subgroup analyses according to the diagnostic criteria of TRL, regression analysis model (univariate or multivariate), and indications of ICIs (for locally advanced or advanced lung cancer) showed consistent results (p for subgroup difference all > 0.05).

**Conclusion:**

TRL was associated with poor survival of lung cancer patients who were treated with ICIs.

## Introduction

Lung cancer is a prevalent malignancy affecting the global population (1). According to the global cancer statistics in 2020, lung cancer constituted 11.4% of all cancer cases and accounted for 18.0% of cancer-related fatalities worldwide (2). Histologically, lung cancer can be categorized as non-small cell lung cancer (NSCLC) and small cell lung cancer (SCLC), with therapeutic interventions primarily encompassing surgery, radiation therapy, chemotherapy, and targeted drug therapy (3, 4). A growing body of research underscores the significance of the immune system in cancer surveillance and anti-tumor activity (5, 6). Recent evidence has emphasized the significant role of immune checkpoint inhibitors (ICIs) as efficacious anticancer agents through the inhibition of programmed death-ligand 1 (PD-L1), programmed death 1 (PD-1), or cytotoxic t-lymphocyte-associated protein 4 (CTLA-4) receptors, thereby augmenting the cytotoxicity of T lymphocytes towards tumor cells (7). The overall effectiveness and safety of immunotherapy utilizing ICIs have been generally demonstrated in patients afflicted with metastatic and locally advanced non-small cell lung cancer (NSCLC) (8), as well as small cell lung cancer (SCLC) (9). However, subsequent observations suggest that the therapeutic response to ICIs may vary in individual patients with lung cancer (10). Accordingly, uncovering of the clinical factors that are related to the efficacy of ICIs in patients with lung cancer is of great clinical significance.

Lymphopenia is a prevalent occurrence in cancer patients, primarily attributed to the administration of anticancer treatments such as radiotherapy and chemotherapy, referred to as treatment-related lymphopenia (TRL) (11, 12). A recent meta-analysis encompassing 14 studies revealed that the average occurrence of severe lymphopenia (defined as an absolute lymphocyte count [ALC] < 500/ul) in lung cancer patients undergoing radiotherapy was 64.2% (13). Although sever TRL has been related to poor prognosis in patients with various solid tumors including lung cancer in early studies, patients with concurrent ICIs were rarely included in these studies (14). The potential impact of TRL on the effectiveness of ICIs, specifically by reducing the active T lymphocytes, has been postulated (15). However, the precise effect of TRL on the efficacy of ICIs in individuals with lung cancer has yet to be fully elucidated. Consequently, we conducted a comprehensive review and meta-analysis to examine the influence of TRL on the survival outcomes of lung cancer patients undergoing ICIs treatment.

## Materials and methods

The study adhered to the Preferred Reporting Items for Systematic Reviews and Meta-Analyses statement (16, 17) and the Cochrane Handbook (18) throughout the stages of planning, conducting, and reporting.

### Inclusion and exclusion criteria of studies

The development of inclusion criteria adhered to the PICOS recommendations and aligned with the objective of the meta-analysis.

P (patients): Patients with pathologically confirmed diagnosis of lung cancer who were treated with ICIs.

I (exposure): Patients with TRL at the initiation or during ICIs treatment. Diagnostic criteria and cutoffs for defining TRL were consistent with those of the original studies. We included studies of patients with lymphopenia related to any anticancer treatment, not limited to those of patients received radiation only.

C (control): Patients without TRL.

O (outcomes): The study compared the progression-free survival (PFS) and/or overall survival (OS) outcomes between individuals with and without TRL. In essence, PFS denotes the time from the initiation of treatment to the occurrence of disease recurrence or progression, while OS represents the time from the initiation of treatment to the patient’s eventual demise.

S (study design): This study incorporated longitudinal follow-up studies, such as cohort and nested case-control studies, along with *post-hoc* analyses of clinical trials. Excluded from the meta-analysis were reviews, editorials, meta-analyses, preclinical studies, and studies that did not involve patients with lung cancer or with ICIs, failed to evaluate TRL, or did not report the survival outcomes of interest during follow-up. In cases where there was an overlap in patient populations, the study with the largest sample size was included in the meta-analysis.

### Search of databases

A comprehensive search was conducted in electronic databases, namely PubMed, Embase, Cochrane Library, and Web of Science, encompassing the period from inception to July 10, 2023. The search strategy employed relevant terms pertaining to the subject matter of our investigation, aiming to identify studies published within this timeframe, which included: (1) “lymphopenia” OR “lymphocytopenia”; (2) “lung cancer”; and (3) “immunotherapy” OR “immune checkpoint inhibitor” OR “PD-1” OR “PD-L1” OR “CTLA-4” OR “programmed death 1” OR “programmed death ligand 1” OR “pembrolizumab” OR “atezolizumab” OR “nivolumab” OR “ipilimumab” OR “durvalumab” OR “tremelimumab” OR “camrelizumab” OR “tislelizumab” OR “sintilimab” OR “cemiplimab” OR “toripalimab” OR “lambrolizumab” OR “pidilizumab” OR “avelumab”. Only studies that met the criteria of being published as full-length articles in English and appearing in peer-reviewed journals were included in our analysis. Additionally, during our manual screening process, we thoroughly examined the references cited in relevant original and review articles to identify any potentially relevant studies.

### Data extraction and quality evaluation

Two authors independently performed literature searches, data collection, and assessments of study quality. In cases where discrepancies emerged, a third author was consulted for deliberation, leading to a consensus. The analysis of studies encompassed the gathering of data related to study information, design characteristics, patient diagnosis, demographic factors, medications for immunotherapy, definition of TRL, number of patients with TRL, median follow-up durations, outcomes reported, and variables adjusted for the evaluation of the association between TRL and survival of lung cancer patients on ICIs. The quality of the study was assessed using the Newcastle-Ottawa Scale (NOS) (19), which evaluates participant selection, group comparability, and outcome validity. The scale consisted of nine stars, with a greater number of stars indicating a study of higher quality.

### Statistics

Hazard ratios (HRs) and their corresponding 95% confidence intervals (CIs) were utilized as the variables to assess the relationship between TRL and the survival of lung cancer patients receiving immune ICIs. To stabilize and normalize the variance, a logarithmical transformation was applied to the HR and its corresponding standard error in each study (20). The Cochrane Q test and the I^2^ statistic (21) were employed to estimate between-study heterogeneity. A value of I^2^ greater than 50% indicates the presence of significant heterogeneity among the studies. The random-effects model was utilized to combine the findings, as it has been recognized to account for potential heterogeneity (18). Sensitivity analysis limited to patients with NSCLC was performed. Additionally, subgroup analysis was conducted to explore the influence of cutoffs for TRL, different regression analysis model (univariate or multivariate), and indications of ICIs (for locally advanced or advanced lung cancer) on the outcomes. The cutoffs for defining overall TRL (<1000 lymphocytes/ul) and severe TRL (<500 lymphocytes/ul) were in accordance with the Common Terminology Criteria for Adverse Events (CTCAE) criteria (22). Publication bias was estimated using a funnel plot, which involved visual assessments of symmetry, as well as Egger’s regression asymmetry test (23). The statistical analyses were conducted using RevMan (Version 5.1; Cochrane Collaboration, Oxford, UK) and Stata software (version 12.0; Stata Corporation, College Station, TX).

## Results

### Database search and study retrieval

**Figure 1:**
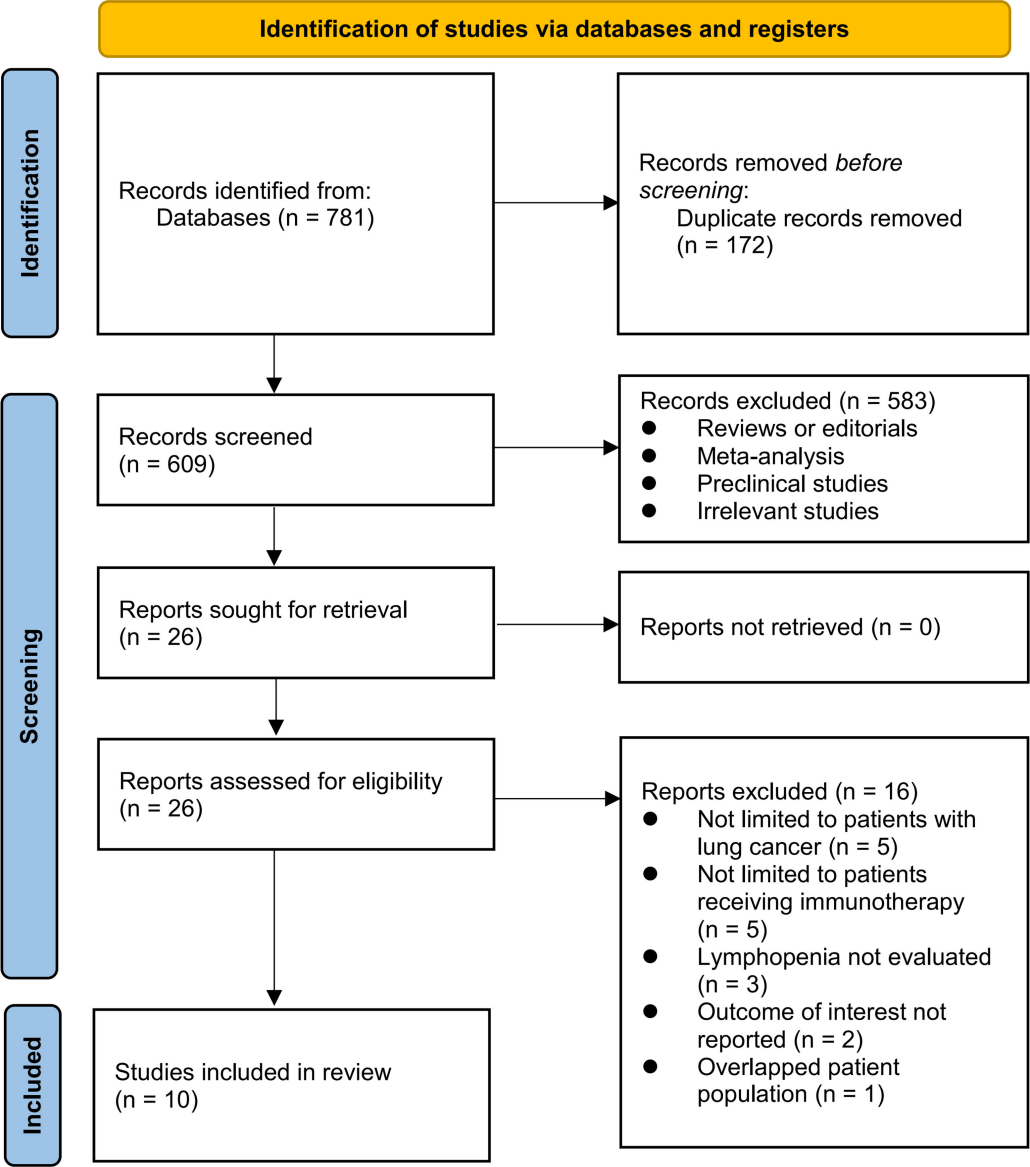
Flowchart of database search and study inclusion.

**Figure 1** illustrates the procedure employed for conducting the literature search and study retrieval. Initially, a total of 781 records were acquired from the designated database, and subsequently, 172 duplicate entries were eliminated. Upon scrutinizing the titles and abstracts, an additional 583 studies were excluded due to their incompatibility with the objectives of the meta-analysis. Following comprehensive evaluations of the full texts of 26 studies, 16 were excluded based on the rationales outlined in **Figure 1**. Consequently, ten studies were deemed suitable for the subsequent meta-analysis (24–33).

### Study characteristics

Overall, one prospective (27) and nine retrospective cohort studies (24–26, 28–33) were included in the meta-analysis. The characteristics of the studies incorporated in this analysis are concisely outlined in **Table 1**. These studies were conducted in the United States, Korea, and France, and were published within the timeframe of 2019 to 2023. All of the studies encompassed patients diagnosed with lung cancer who underwent treatment with ICIs. Among these studies, eight exclusively focused on patients with NSCLC (24, 25, 28–33), whereas the remaining two also encompassed patients with SCLC (26, 27). The drugs for ICIs varied among the included studies, which involved nivolumab, pembrolizumab, durvalumab, atezolizumab, ipilimumab, or a combination of nivolumab and ipilimumab. The cutoffs for the diagnosis of TRL also varied among the included studies, and accordingly, 427 patients (37.8%) were diagnosed as TRL. The follow-up durations varied from 4.7 months to 24.0 months, with the median follow-up duration of 13.0 months. Outcome of PFS was reported in nine studies (24, 26–33), while the outcome of OS was reported in eight studies (24–27, 29–31, 33). Univariate regression analysis was used in three studies when the association between TRL and survival of lung cancer patients on ICIs was reported (25, 26, 30), while in the other seven studies (24, 27–29, 31–33), multivariate regression analysis was used with the adjustment of confounding factors such as age, sex, performance status, histological type of cancer, and other concurrent anticancer treatments etc. The NOS of the included studies were six to nine, indicating that they were of moderate to good quality (**Table 2**).

**Table 1:** Characteristics of the included studies.

Study	Country	Design	Diagnosis	Number of patients	Mean age (years)	Male (%)	ICIs used	Definition of lymphopenia	Number of patients with lymphopenia	Median follow-up duration (months)	Outcomes reported	Variables adjusted
Karantanos 2019 (25)	USA	RC	Advanced NSCLC	22	62	54.5	Nivolumab	ALC < 900/ul at baseline or 6 months after the initiation of ICIs	6	10	OS	None
Cho 2019 (24)	Korea	RC	Advanced NSCLC	268	64	67.9	Nivolumab, pembrolizumab, or a combination of nivolumab and ipilimumab	ALC < 1000/ul at baseline or during ICIs	146	6.4	PFS and OS	Age, sex, ECOG PS, histological type, PD-L1 expression, EGFR mutation, disease extent, previous treatment, use or RT, and medications of ICIs
Li 2019 (26)	USA	RC	Lung cancer patients with brain metastases (NSCLC 93.6%)	20	65	46	Any ICIs	ALC < 1000/ul at baseline or during ICIs	15	13.2	PFS and OS	None
Chen 2020 (27)	USA	PC	Lung cancer patients receiving combined immunotherapy and radiotherapy (NSCLC 36.4%)	165	65	56.4	Ipilimumab or pembrolizumab	ALC < 1300/ul at baseline or during ICIs	69	21	PFS and OS	Age, sex, race, ECOG PS, histological type, smoking, prior treatment, and medications of ICIs
Friedes 2021 (28)	USA	RC	Unresectable locally advanced NSCLC	78	66	55	Durvalumab or Ipilimumab + nivolumab	ALC < 500/ul at the initiation of ICIs	18	10.6	PFS	Age, sex, race, KPS, tumor size, previous treatment, and medications of ICIs
Jing 2022 (30)	USA	RC	Locally advanced NSCLC	117	NR	59	Durvalumab	ALC < 230/ul at baseline or during ICIs	28	24	PFS and OS	None
Cho 2022 (29)	Korea	RC	Stage III NSCLC	66	65	82	Durvalumab or pembrolizumab	ALC < 500/ul at the initiation and during ICIs	7	18	PFS and OS	Age, sex, histological type, PD-L1 expression, and medications of ICIs
Thor 2022 (32)	USA	RC	Stage III NSCLC	113	67	59	Durvalumab	ALC < 700/ul at the initiation of ICIs	56	17.5	PFS	Age, ECOG PS, and PD-L1 expression
Lee 2022 (31)	Korea	RC	Advanced NSCLC	231	66	78.5	Durvalumab, pembrolizumab, or atezolizumab	ALC < 1000/ul at baseline or during ICIs	57	4.7	PFS and OS	Age, sex, ECOG PS, histological type, and smoking
Pasquier 2023 (33)	France	RC	Unresectable locally advanced NSCLC	50	61.5	76	Durvalumab	ALC < 500/ul at the initiation of ICIs	25	23.2	PFS and OS	Age, sex, ECOG PS, histological type, and smoking

RC, retrospective cohort; PC, prospective cohort; NSCLC, non-small cell lung cancer; ICIs, immune checkpoint inhibitors; ALC, absolute lymphocyte count; PFS, progression-free survival; OS, overall survival; ECOG PS, the Eastern Cooperative Oncology Group Performance Status; PD-L, programmed death ligand 1; EGFR, epidermal growth factor receptor; RT, radiotherapy; KPS, Karnofsky performance status.

**Table 2:** Study quality assessment via the Newcastle-Ottawa Scale.

Study	Representativeness of the exposed cohort	Selection of the non-exposed cohort	Ascertainment of exposure	Outcome not present at baseline	Control for age	Control for other confounding factors	Assessment of outcome	Enough long follow-up duration	Adequacy of follow-up of cohorts	Total
Karantanos 2019 (25)	0	1	1	1	0	0	1	1	1	6
Cho 2019 (24)	0	1	1	1	1	1	1	1	1	8
Li 2019 (26)	0	1	1	1	0	0	1	1	1	6
Chen 2020 (27)	1	1	1	1	1	1	1	1	1	9
Friedes 2021 (28)	0	1	1	1	1	1	1	1	1	8
Jing 2022 (30)	0	1	1	1	0	0	1	1	1	6
Cho 2022 (29)	0	1	1	1	1	1	1	1	1	8
Thor 2022 (32)	0	1	1	1	1	1	1	1	1	8
Lee 2022 (31)	0	1	1	1	1	1	1	0	1	7
Pasquier 2023 (33)	0	1	1	1	1	1	1	1	1	8

### Influence of TRL on PFS in lung cancer patients treated with ICIs

**Figure 2:**
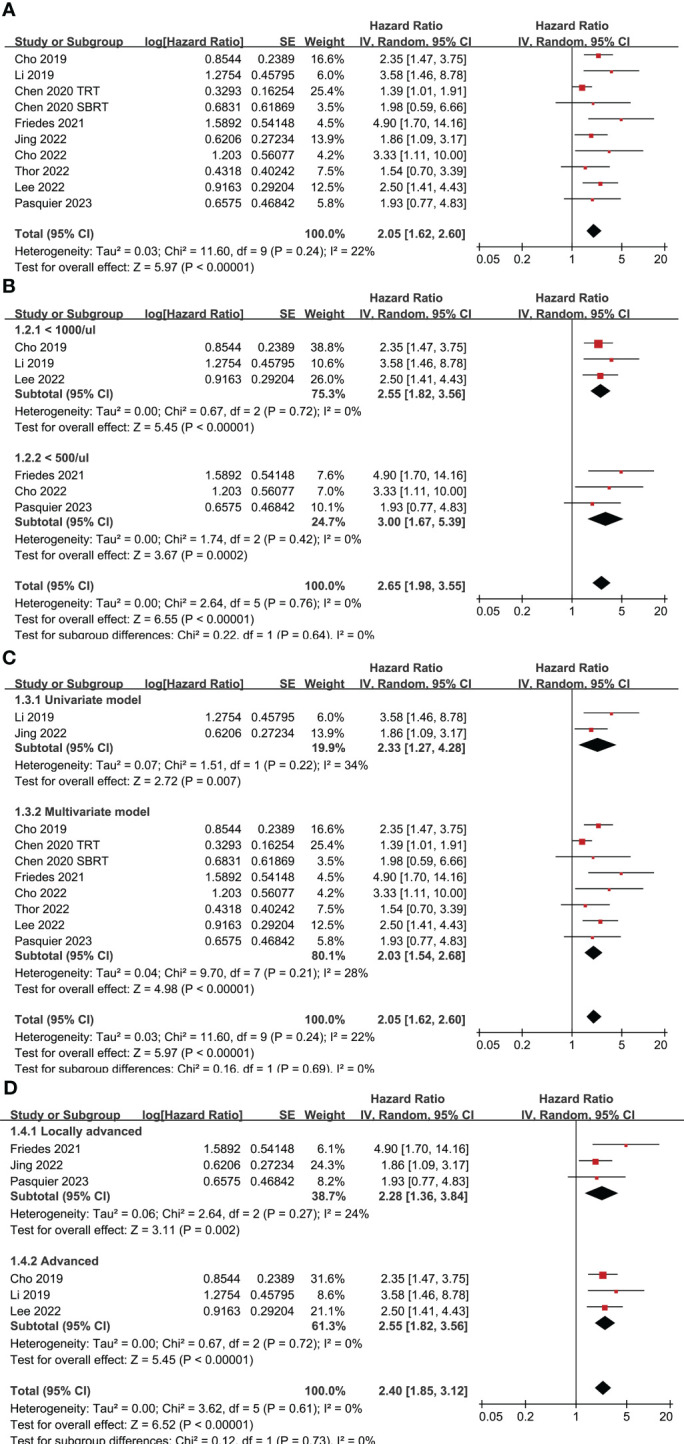
Forest plots for the meta-analyses regarding the association between TRL and PFS of lung cancer patients on ICIs; **(A)** overall meta-analysis; **(B)** subgroup analysis according to the cutoff for the diagnosis of TRL; **(C)** subgroup analysis according to the different analytic models used in the original studies; and **(D)** subgroup analysis according to the indications of ICIs.

Nine studies (24, 26–33) evaluated the association between TRL and PFS in lung cancer patients treated with ICIs. Since one study reported the outcome in two cohorts of patients with different concurrent radiotherapy strategies (27), these two datasets were included independently into the meta-analysis. Pooled results showed that compared to those without TRL, lung cancer patients with TRL was associated with poor PFS (HR: 2.05, 95% CI: 1.62 to 2.60, p < 0.001; **Figure 2A**) with mild heterogeneity (I^2 = ^22%). Sensitivity analysis limited to patients with NSCLC showed similar results (HR: 2.26, 95% CI: 1.76 to 2.91, p < 0.001; I^2 = ^0%). Further subgroup analysis showed consistent association between TRL and poor PFS in studies with TRL diagnosed as ALC < 1000 and < 500/ul (p for subgroup difference = 0.64; **Figure 2B**), in studies with univariate and multivariate analysis (p for subgroup difference = 0.69; **Figure 2C**), and in studies with ICIs for locally advanced or advanced lung cancer (p for subgroup difference = 0.73; **Figure 2D**).

### Influence of TRL on OS in lung cancer patients treated with ICIs

**Figure 3:**
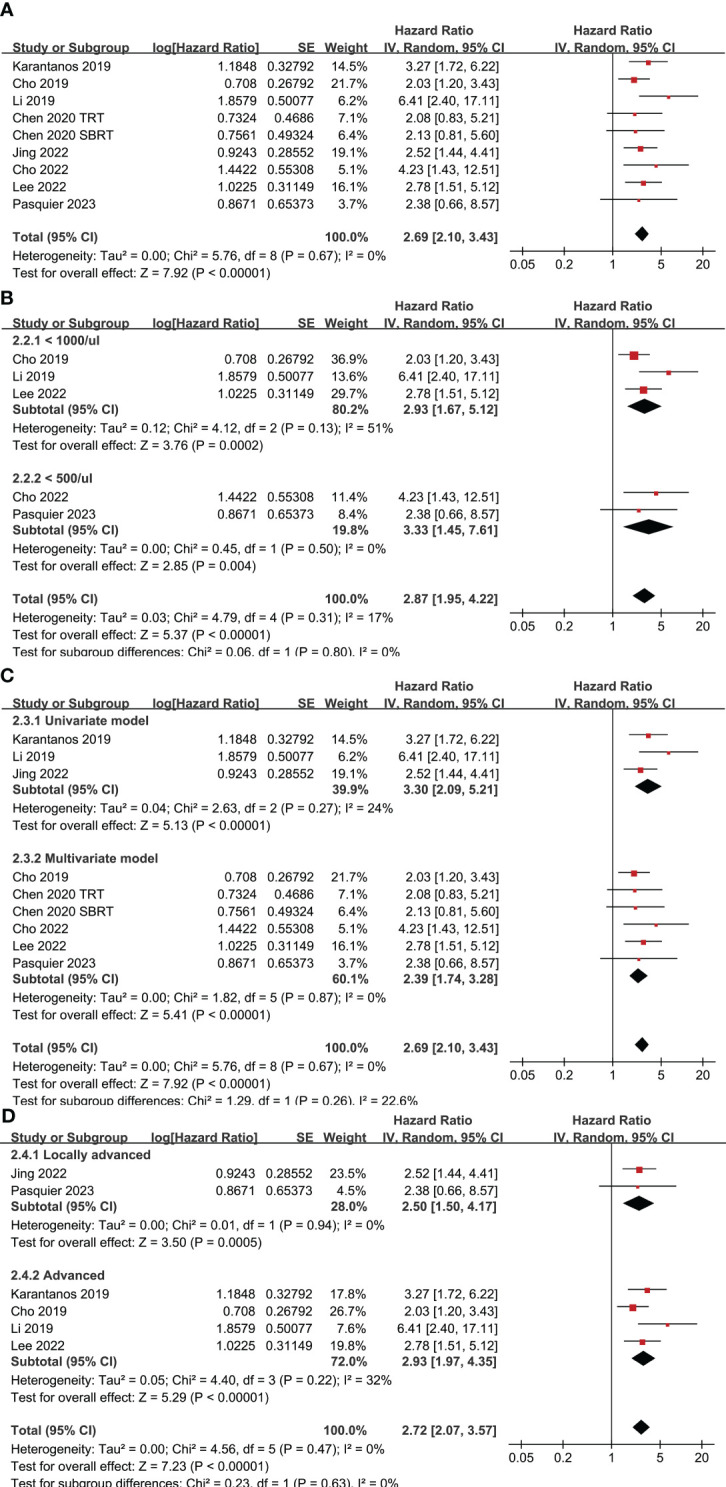
Forest plots for the meta-analyses regarding the association between TRL and OS of lung cancer patients on ICIs; **(A)** overall meta-analysis; **(B)** subgroup analysis according to the cutoff for the diagnosis of TRL; **(C)** subgroup analysis according to the different analytic models used in the original studies; and **(D)** subgroup analysis according to the indications of ICIs.

Eight studies (24–27, 29–31, 33) with nine datasets were included for the meta-analysis of the association between TRL and OS in lung cancer patients on ICIs. Results of the meta-analysis showed that TRL was associated with poor OS (HR: 2.69, 95% CI: 2.10 to 3.43, p < 0.001; **Figure 3A**) with no significant heterogeneity (I^2 = ^0%). Consistent results were observed in sensitivity analysis limited to NSCLC only (HR: 2.62, 95% CI: 1.99 to 3.44, p < 0.001; I^2 = ^0%). Moreover, subgroup analyses according to the diagnostic criteria of TRL (p for subgroup difference = 0.80, **Figure 3B**), the analytic models (p for subgroup difference = 0.26, **Figure 3C**), and indications of ICIs (p for subgroup difference = 0.63, **Figure 3D**) also showed similar results.

### Publication bias

**Figure 4:**
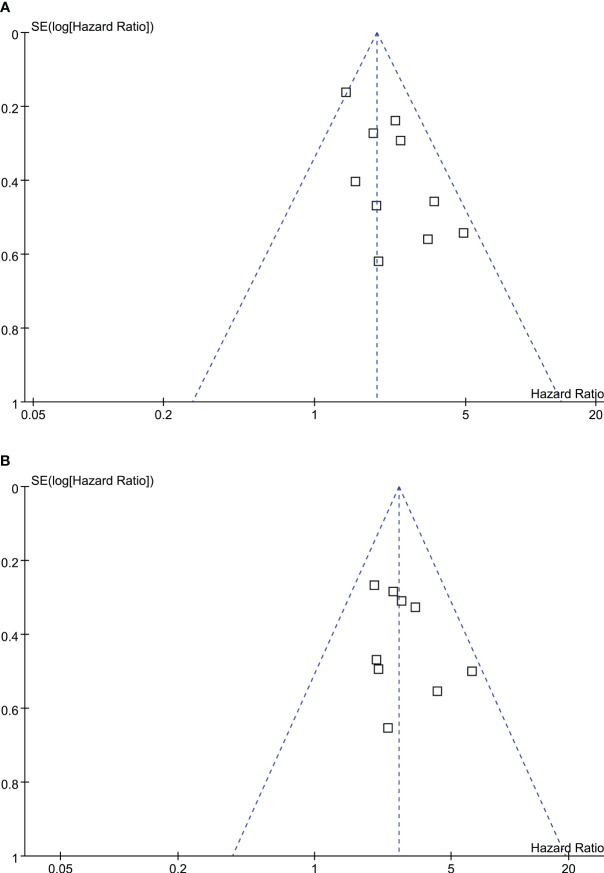
Funnel plots for the publication bias underlying the meta-analyses of the association between TRL and survival outcomes of lung cancer patients on ICIs; **(A)** funnel plots for the meta-analysis of the association between TRL and PFS; and **(B)** funnel plots for the meta-analysis of the association between TRL and OS.

The funnel plots depicting the meta-analyses of the correlation between TRL and survival outcomes among lung cancer patients receiving ICIs are presented in **Figure 4**. Upon visual inspection, the plots exhibit symmetrical patterns, indicating a minimal presence of publication bias. Furthermore, the application of Egger’s regression tests yielded p-values of 0.37 and 0.49, further supporting the notion of a low probability of publication bias.

## Discussion

This study conducted a systematic review and meta-analysis, incorporating data from ten cohort studies, to examine the correlation between TRL and survival outcomes in lung cancer patients undergoing ICIs treatment. The results of our analysis suggest that the presence of TRL at the start or during ICIs treatment is linked to unfavorable PFS and OS in lung cancer patients. Additionally, when focusing solely on studies involving patients with NSCLC, the findings remained consistent. Furthermore, subgroup analyses based on the cutoff value for diagnosing TRL, the chosen analytical model, and indications of ICIs also yielded consistent results. Taken together, these results suggest that TRL may be a risk factor of poor survival of lung cancer patients on the treatment of ICIs.

To the best of our knowledge, this study represents a potentially pioneering meta-analysis that examines the impact of TRL on the effectiveness of ICIs in individuals diagnosed with lung cancer. It is worth highlighting several notable advantages inherent in the employed meta-analysis methodologies. For instance, an extensive search of four widely utilized databases was conducted, thereby yielding up-to-date evidence pertaining to the association between TRL and the survival outcomes of lung cancer patients undergoing ICIs treatment. Moreover, it is worth mentioning that all the studies included in this analysis were cohort studies, indicating a possible longitudinal association between TRL and heightened risk of disease progression and mortality in these individuals. Furthermore, subgroup analysis based on the threshold values of ALC for diagnosing TRL yielded consistent findings, implying that even a mild TRL of ALC < 1000/ul may have a detrimental impact on the prognosis of lung cancer patients receiving ICIs. Finally, consistent results were obtained for subgroups of univariate and multivariate regression analyses, which suggested that the association between TRL and poor survival of lung cancer patients on ICIs may be independent of variables such as age, sex, functional status, and previous anticancer treatments. Collectively, these findings highly suggest the importance of monitoring lymphocyte count in peripheral circulating during the treatment with ICIs for patients with lung cancer.

The negative consequences observed in patients experiencing lymphopenia may be ascribed to modifications in the tumor microenvironment. These alterations can be linked to the accumulation of myeloid-derived suppressor cells, type-2 macrophages, or regulatory T cells, along with the generation of suppressive cytokines and metabolites, which can foster tumor advancement (15). Additionally, it has been postulated that neoantigen-specific T cells can be detected in the peripheral blood of patients with NSCLC undergoing anti-PD-L1 therapy. Notably, patients who exhibited an objective response demonstrated an increased presence of neoantigen-reactive T cells, which exhibited distinct phenotypic characteristics compared to non-responsive patients (34). The migration of these T cells to metastatic sites following immune checkpoint blockade stimulation is believed to play a crucial role in eliciting an effective antitumor response. However, the inhibition of T-cell function and subsequent lymphopenia in the peripheral blood may impede the transfer of T cells to the tumor site. In this particular scenario, lymphopenia serves as a surrogate marker indicating resistance to ICIs, necessitating the need for treatment modifications to overcome this resistance (35, 36). Notably, a recent investigation involving lung cancer patients with TRL who received ICIs revealed that inadequate lymphocyte recovery correlated with a shorter PFS, an increase in regulatory T cells, and a depletion of CD8+ T cells in the peripheral blood. These findings suggest that prompt recovery from TRL may hold significance in enhancing the prognosis of these patients (37).

For patients with cancer, radiation is a common cause of TRL, which is called radiation-induced lymphopenia (RIL). In lung cancer patients, the risk factors of RIL include advanced age, ALC before treatment, higher mean lung dose, larger volume of lung and heart receiving low dose (V5), longer treatment duration, and longer total beam-on time etc. (38–40). Among the studies included in the current meta-analysis, TRL caused by radiation therapy most frequently occurred at or within the six months after the initiation of the ICIs therapy. Results of the meta-analysis suggested that TRL may be associated with poor survival of lung cancer patients receiving ICIs, which is consistent with the results of studies in other types of tumors. A previous study of 105 patients with recurrent metastatic esophageal cancer receiving immunotherapy showed that lymphopenia is associated with a poorer immunotherapy prognosis in these patients (41). In another study of patients treated with nivolumab for recurrent/metastatic head and neck cancer, head and neck cancer, persistence of lymphopenia during immunotherapy was shown to be a predictor of worse OS (42). One attractive question at current stage is whether a prompt recovery from TRL may hold significance in enhancing the prognosis of patients with lung cancer on ICIs. A recently published pilot observational study in stage III NSCLC patients undergoing durvalumab consolidation therapy have suggested that recovery from TRL at the initiation of ICIs were associated with improved PFS and OS as compared to those without lymphocyte recovery (43). From a clinical standpoint, the restriction of radiotherapy dosage to the lungs, heart, and vertebrae presents modifiable risk factors that could potentially decrease the occurrence of RIL and PFS and OS, particularly in patients with non-modifiable risk factors such as advanced age, lower pre-radiotherapy ALC, and larger tumor size. However, modifying Lung V5 may pose technical challenges as it could lead to an increase in V20, thereby exacerbating fibrosis. Alternatively, shorter treatment duration may prove to be a more effective strategy when combined with immunotherapy to mitigate lymphopenia (44). Moreover, in the case of metastatic lung cancers, the utilization of stereotactic body radiotherapy to irradiate a specific portion of the tumor, as opposed to conventional radiotherapy targeting the entire tumor, has the potential to reduce RIL, while simultaneously preserving lung function and promoting antigen release. This, in turn, can contribute to the abscopal response (45). To comprehensively comprehend the relationship between TRL and unfavorable survival, as well as to devise efficacious approaches for improving the prognosis of patients experiencing lymphopenia during ICIs therapy, further translational investigations and clinical trials are imperative.

This study has several limitations that should be acknowledged. Firstly, the protocol of the meta-analysis was not prospectively registered, which is acknowledged as a limitation. Secondly, majority of the studies included in this analysis were retrospective, which introduces the possibility of selection and recall biases. To validate the findings, it is necessary to conduct large-scale prospective studies. Thirdly, among eight of the ten included studies, patients with NSCLC were included, while the other two studies included patients with NSCLC and SCLC. Accordingly, we could only observe the association between TRL and survival in NSCLC patients with ICIs via a sensitivity analysis limited to studies of patients with NSCLC only, instead of a subgroup analysis of NSCLC versus SCLC. It is important to considering subgroup analysis in NSCLC versus SCLC, because compared to NSCLC, SCLC is characterized by an exceptionally high proliferative rate, strong tendency for early widespread metastasis, and acquired chemoresistance (46). Currently, we could not determine if the association between TRL and survival of patients with SCLC on ICIS could be different from those of NSCLC, and studies are needed to address this association in patients with SCLC in the future. Furthermore, although the subset of studies employing multivariate regression analyses yielded comparable findings, it is important to acknowledge that the influence of residual factors on the correlation between TRL and unfavorable survival outcomes in these patients cannot be definitively dismissed. Ultimately, due to the nature of this meta-analysis being based on observational studies, it is not possible to establish a causal relationship between TRL and the heightened risk of cancer progression and mortality in lung cancer patients undergoing ICIs. Therefore, it is imperative to conduct clinical studies to ascertain if effective prevention or recovery of TRL could favorably influence the survival of lung cancer patients with ICIs treatment.

## Conclusions

The results of the meta-analysis suggest a correlation between TRL and decreased survival rates among lung cancer patients receiving ICIs, particularly in those with NSCLC. However, further prospective studies are necessary to confirm these findings. On the other hand, the meta-analysis underscores the significance of monitoring peripheral lymphocyte counts in lung cancer patients undergoing ICI treatment. Additionally, it is crucial to investigate whether interventions aimed at preventing or expediting the recovery of TRL are linked to improved survival outcomes for lung cancer patients undergoing ICIs treatment.

## Data availability statement

The original contributions presented in the study are included in the article/supplementary material. Further inquiries can be directed to the corresponding author.

## Author contributions

YZ: Conceptualization, Data curation, Formal Analysis, Investigation, Methodology, Writing – original draft. CH: Data curation, Formal Analysis, Investigation, Validation, Writing – review & editing. SL: Conceptualization, Data curation, Formal Analysis, Investigation, Methodology, Supervision, Validation, Writing – review & editing.
